# Development of an embryonic skeletogenic mesenchyme lineage in a sea cucumber reveals the trajectory of change for the evolution of novel structures in echinoderms

**DOI:** 10.1186/2041-9139-3-17

**Published:** 2012-08-09

**Authors:** Brenna S McCauley, Erin P Wright, Cameron Exner, Chisato Kitazawa, Veronica F Hinman

**Affiliations:** 1 Department of Biological Sciences, Carnegie Mellon University, 4400 5th Avenue, Pittsburgh, PA, 15213, USA

**Keywords:** Sea cucumber, Echinoderm, Evolution of novelty, Co-option, Skeletogenesis, Alx1

## Abstract

**Background:**

The mechanisms by which the conserved genetic “toolkit” for development generates phenotypic disparity across metazoans is poorly understood. Echinoderm larvae provide a great resource for understanding how developmental novelty arises. The sea urchin pluteus larva is dramatically different from basal echinoderm larval types, which include the auricularia-type larva of its sister taxon, the sea cucumbers, and the sea star bipinnaria larva. In particular, the pluteus has a mesodermally-derived larval skeleton that is not present in sea star larvae or any outgroup taxa. To understand the evolutionary origin of this structure, we examined the molecular development of mesoderm in the sea cucumber, *Parastichopus parvimensis*.

**Results:**

By comparing gene expression in sea urchins, sea cucumbers and sea stars, we partially reconstructed the mesodermal regulatory state of the echinoderm ancestor. Surprisingly, we also identified expression of the transcription factor *alx1* in a cryptic skeletogenic mesenchyme lineage in *P. parvimensis*. Orthologs of *alx1* are expressed exclusively within the sea urchin skeletogenic mesenchyme, but are not expressed in the mesenchyme of the sea star, which suggests that *alx1*^+^ mesenchyme is a synapomorphy of at least sea urchins and sea cucumbers. Perturbation of Alx1 demonstrates that this protein is necessary for the formation of the sea cucumber spicule. Overexpression of the sea star *alx1* ortholog in sea urchins is sufficient to induce additional skeleton, indicating that the Alx1 protein has not evolved a new function during the evolution of the larval skeleton.

**Conclusions:**

The proposed echinoderm ancestral mesoderm state is highly conserved between the morphologically similar, but evolutionarily distant, auricularia and bipinnaria larvae. However, the auricularia, but not bipinnaria, also develops a simple skelotogenic cell lineage. Our data indicate that the first step in acquiring these novel cell fates was to re-specify the ancestral mesoderm into molecularly distinct territories. These new territories likely consisted of only a few cells with few regulatory differences from the ancestral state, thereby leaving the remaining mesoderm to retain its original function. The new territories were then free to take on a new fate. Partitioning of existing gene networks was a necessary pre-requisite to establish novelty in this system.

## Background

It has been known for many years that most animals rely on the use of the same basic set of regulatory genes during development [1]. The great challenge is to determine how novel structures are specified by orthologous genes. Gene regulatory networks (GRNs) for novel structures can presumably be built “from scratch” (also termed *de novo* construction), or be re-wired from existing GRNs, which can include the co-option and fusion of GRN subcircuits. In particular, co-option, that is, the reuse of ancestral regulatory programs in novel contexts, might be a major mechanism through which novelties arise during evolution (reviewed in [2]). For example, this process is thought to have occurred in the formation of butterfly eyespots, patterning appendage outgrowth, the formation of novel insect appendages, and in the evolution of gnathostome oral teeth, among many others [3-8]. Relevant to this study, it has also been suggested that the sea urchin larval skeleton might have arisen via co-option of an adult echinoderm skeletogenic program [9]. However, it is essentially unknown how an ancestral GRN can be rewired to accommodate the acquisition of a co-opted regulatory program in order to produce a viable new state.

The larvae of echinoderms provide a rich source of morphological variation for understanding the evolution of novelty. Broadly, echinoderms have two types of feeding larvae: the pluteus-like larvae of sea urchins and brittle stars, and the auricularia-like larvae of sea cucumbers and sea stars [10]. Interestingly, most sea lilies, which are the earliest-branching echinoderms [11], have a secondarily-derived non-feeding larva, but at least one species is known to go through an auricularia-like stage [12]. Due to the similarity between the hemichordate tornaria larva and the echinoderm auricularia-type larva, this is considered to be the basal form [13], possibly to the whole deuterostome clade. Intriguingly, larval morphology does not track with phylogenetic relationships among the echinoderms. Sea urchins and sea cucumbers are sister taxa, while brittle stars either form the outgroup to this clade or are the sister group to sea stars, though the exact phylogeny remains unclear due to the rapid divergence of these classes [11,14,15]. Sea lilies are thought to be the most basal echinoderms. Thus, the derived plutei of sea urchins and brittle stars do not group together, but instead are scattered among the ancestral auricularia-like larvae in the echinoderm phylogenetic tree.

There are many differences between echinoderm larval forms, but perhaps the most dramatic and obvious is the larval skeleton that is found in the sea urchin plutei that is not at all present in sea star larvae. The larval skeleton provides the structure that gives the sea urchin the dramatic pluteus morphology. The larval skeleton in modern sea urchins (non-irregular euechinoids, hereafter referred to as simply “sea urchins”) forms from the skeletogenic mesenchyme (SM) that arises from micromeres at the vegetal-most pole (reviewed in [16]). The remaining mesoderm goes on to produce mesenchymal blastocoelar cells, as well as pigment cells, epithelial coeloms and muscle [17,18]. The specification of the mesodermal territories is extraordinarily well known in sea urchins, and the SM in particular has, perhaps, the best characterized GRN for any developmental system [19]. Each territory expresses a unique complement of transcription factors during blastula stages, though there is considerable overlap in the expression of individual factors among these lineages. *alx1*, *erg*, *ets1*, *foxn2/3*, *hex*, *tbr* and *tgif* are expressed in the SM lineage [20-27]. The dorsally-restricted pigment cell precursors express *gcm*, *foxn2/3* and *gata4/5/6*[26,28,29], while the ventrally-localized presumptive blastocoelar cells express *erg*, *gata1/2/3*, *foxn2/3*, *ets1* and *gata4/5/6*[25,26,29,30]. Each of these mesodermal cell types goes on to exhibit distinct behavior during gastrulation. The SM ingresses into the blastocoel at the onset of gastrulation and migrates to form a characteristic ring at the dorsal side of the embryo, which terminates in two ventro-lateral clusters. During late gastrulation, cells of the SM fuse and begin secreting the larval skeleton. The pigment cells are the next population of mesenchyme to ingress from the tip of the archenteron during early gastrulation; they migrate to the dorsal ectoderm, into which they intercalate and begin secreting pigment. Blastocoelar cells do not ingress until the late gastrula stage [31].

Previous studies have revealed that orthologs of many of the regulatory genes expressed in the sea urchin mesodermal territories are expressed in the presumptive mesoderm of sea stars [32-34], which will give rise to blastocoelar cells and epithelial mesoderm, but not an SM lineage. We, therefore, undertook an analysis of the expression of orthologous transcription factors in the embryos of a phylogenetic intermediate, the sea cucumber, *Parastichopus parvimensis*, to better correlate changes in regulatory gene expression with morphological novelty. This study is the first comprehensive gene expression analysis performed in any species of sea cucumber and identifies a highly conserved pleisiomorphic mesodermal regulatory state. We show for the first time that sea cucumbers develop a mesodermally-derived skeletogenic mesenchyme from the vegetal pole of blastulae, although the resulting larval skeleton is much less morphologically complex than that of sea urchins*.* Our results suggest that the evolution of larval skeletogenic cells in echinoderms occurred in a step-wise manner, with just minor changes in the regulatory state of the mesodermal precursors occurring prior to subdivisions of the ancestral gene regulatory network subcircuits.

## Results and discussion

### The development of the sea cucumber, ***Parastichopus parvimensis***

Although no sea cucumber has been developed as a model organism, several have been the subject of embryological studies (reviewed in [10]). Several papers reported the development of *Stichopus* (now referred to as *Apostichopus*, *Parastichopus* and *Stichopus*) species [35-37]. In particular, the early development of *S. tremulus* was previously described [35]. Early development in *S. tremulus* is very similar to what we observe in *P. parvimensis* (shown in Additional file 1). Early cleavage is equal and little cell-cell adhesion is seen between blastomeres. Blastulae are formed by 16 hours after fertilization and hatch from the fertilization envelope around 26 hours. Prior to gastrulation, the embryos elongate along the animal-vegetal axis, and a thickening is observed at the vegetal pole, termed the vegetal plate. Mesenchyme cells ingress from the vegetal plate before invagination of the archenteron occurs. At the mid-gastrula stage around 48 hours of development, the mesenchyme has migrated. By 72 hours of development, the mouth has formed, and the embryo is now an early auricularia larva. Presumptive muscle cells are seen associated with the foregut, and a thickened ciliary band is evident in the oral hood and looping above the anus. A small skeletal element is situated at the posterior end of the larva, under the posterior coleom.

### The ancestral echinoderm mesodermal regulatory state

**Figure 1:**
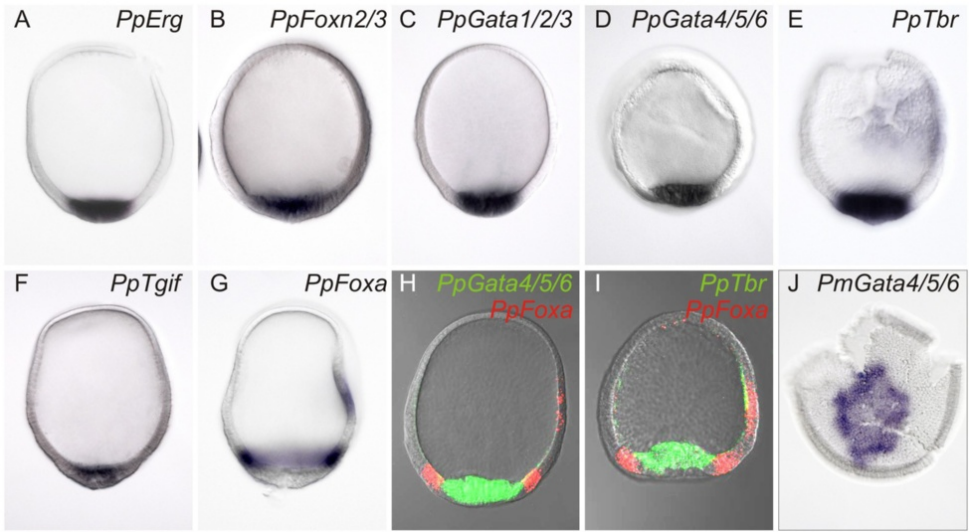
**Orthologous genes are expresed in the mesoderm of sea cucumbers, sea stars and sea urchins.** Whole mount *in situ* hybridization (WMISH) using antisense DIG-labeled probes revealed that sea cucumber orthologs of *erg* (**A**), *foxn2/3* (**B**), *gata1/2/3* (**C**), *gata4/5/6* (**D**), *tbr* (**E**) and *tgif* (**F**) are expressed throughout the mesoderm of sea cucumbers. We also examined the expression of *foxa*, which is expressed in the endoderm of many animals. (**G**) *foxa* is expressed in a ring that likely also marks the endodermally-fated tissue in sea cucumbers. Two-color fluorescent *in situs* with the endoderm marker *foxa* confirm that *gata4/5/6* (**H**) and *tbr* (**I**) are expressed within the *foxa* domain and, therefore, are likely expressed throughout the mesoderm. WMISH also shows that sea star ortholog of *gata4/5/6*, *PmGata4/5/6*, is expressed within the central mesodermal fated territory (**J**).

We sought to determine the commonalities in mesodermal regulatory state that exist between sea stars, sea urchins and sea cucumbers. The transcription factors *erg*, *alx1*, *foxa*, *foxn2/3*, *gata1/2/3*, *gata4/5/6*, *tbr* and *tgif* (named here *PpErg*, *PpAlx1*, *PpFoxa*, *PpFoxn2/3*, *PpGata1/2/3*, *PpGata4/5/6*, *PpTbr* and *PpTgif*, respectively) were selected for this study as these are the orthologs of regulatory genes that operate within the well characterized sea urchin GRN for mesodermal tissue specification. Phylogenetic analysis confirms the orthology of each gene (Additional file 2). *PpErg*, *PpFoxn2/3*, *PpGata1/2/3*, *PpGata4/5/6*, *PpTbr* and *PpTgif* are all expressed throughout the presumptive mesoderm at the central vegetal pole of sea cucumber embryos (Figure 1A-F). We confirmed this using a series of two-color *in situ* hybridizations, showing, for instance, that both *PpTbr* and *PpGata4/5/6* are expressed throughout a territory that is bounded by *PpFoxa* expression (Figure 1G-I). *foxa*, which is expressed in a torus above the central vegetal plate, likely marks the ring of future endoderm in sea cucumbers as it does in sea urchins and sea stars [33,38]. It has also previously been shown that the *ets1* transcription factor is expressed in the vegetal pole of a closely-related species of sea cucumber [39]. Expression of sea star orthologs of these transcription factors has been reported [32-34,40]. We re-examined the expression of the sea star *PmGata4/5/6* gene. At an early blastula stage, *PmGata4/5/6* is detected in the central vegetal pole mesodermal precursors (Figure 1J), which was not previously reported [40].

Therefore, the sea cucumber, like the sea star, forms a central domain of mesodermally fated cells in which orthologs of *erg*, *ets1*, *gata4/5/6*, *foxn2/3*, *tbr*, *tgif* and *gata1/2/3* are co-expressed. As such, these transcription factors, at least, represent the core set of regulatory genes that has been maintained in a single mesodermal domain of blastulae since sea cucumbers and sea stars last shared a common ancestor some 450 million years ago [11]. This suggests that there has been strong selection for this mesodermal regulatory state in the auricularia-like larvae of these two classes of echinoderms. This pleisiomorphic mesoderm is a mixture of the regulatory states that are now segregated into separate mesodermal lineages in modern sea urchins. Of course, transcription factors uniquely expressed in the mesoderm of one organism or another have been identified and more are likely to be found.

### The sea cucumber, like the sea urchin, has an SM lineage that arises from the mesoderm during early development

**Figure 2:**
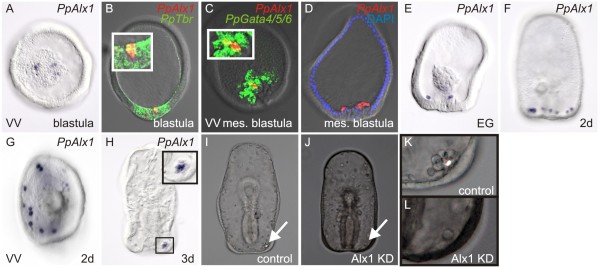
**The sea cucumbers skeletogenic mesenchyme arises from the vegetal pole mesoderm during early development.** WMISH shows that sea cucumber *alx1* transcripts are localized to a population of four cells in the vegetal pole of blastulae (**A**). During this stage, *PpAlx1* is co-expressed with other factors in the mesoderm, as shown here by two-color fluorescent *in situ* with probes antisense to *PpTbr* (**B**) and *PpGata4/5/6* (**C**). Inset is higher magnification view (B and C). The *PpAlx1*^+^ cells are among the earliest to ingress at the onset of gastrulation (**D**). During gastrulation, PpAlx transcripts are localized to the first cells to migrate away from the archenteron (**E**) and transcripts are later localized in a ring of cells on the dorsal side of the embryo (**F, G**). In larvae, *alx1* transcripts are detected specifically in a localized cluster of cells that corresponds to the position of the skeletal spicule spicule (**H**; cell cluster is inset). Knockdown of PpAlx1 results specifically in the loss of the skeletal element (arrows) without drastic changes in larval morphology (**I** vs. **J**; high magnification shown in **K** and **L**). mes. blastula - mesenchyme blastula; EG-early gastrula; d-day; vv-vegetal pole view; control - control MASO injected; KD – MASO knock down.

Despite the overall similarity of regulatory gene expression between the sea cucumber and sea star, we show for the first time that the sea cucumber also develops an SM lineage and that this population is most likely homologous to the sea urchin SM lineage. It has been known for some time that sea cucumber larvae later produce a small spicule, morphologically quite unlike the sea urchin larval skeleton [10], but the embryonic origin of this skeletal element has not been determined. In sea urchins, *SpAlx1* is one of the first transcription factors activated within the SM lineage and is needed for the specification of these cells [21]. In sea cucumber blastulae, *PpAlx1* is expressed in just four cells in the vegetal pole mesoderm (Figure 2A). Initially, this gene is co-expressed with other TFs expressed in the mesoderm, as shown by two-color *in situ* hybridization with *PpTbr *and *PpGata4/5/6* (Figure 2B, C). The *PpAlx1*^+^ cells are among the first to ingress at the onset of gastrulation and are the first to migrate away from the archenteron (Figure 2D, E). These cells form a chain on the dorsal side of the embryo (Figure 2F, G), much like that seen with the sea urchin SM, and then are found as a cluster of approximately eight cells localized underneath the dorsal ectoderm (Figure 2H). This cell cluster appears to be in the same location as the skeletal spicule. To further confirm that the *PpAlx1*^+^ cells are the skeletogenic cells of sea cucumber larvae, we inhibited translation of PpAlx1 by injection of an antisense morpholino-substituted oligonucleotide. As shown in Figure 2I-L, such morphants are morphologically very normal, except they specifically lack the cluster of cells that secretes the spicule. Thus, phenomenologically, the behavior of the *PpAlx1*-expressing cells in sea cucumbers is extraordinarily similar to that of the *SpAlx1*-expressing SM in sea urchins. Both populations undergo early ingression, directed dorsal migration to form a ring of cells, and cluster to secrete the larval skeleton. We conclude that the *PpAlx1*-expressing cells in sea cucumbers are homologous to the SM lineage of sea urchins.

**Figure 3:**
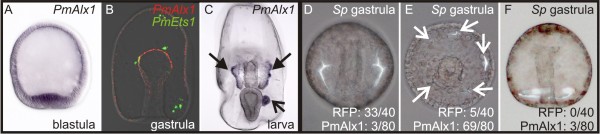
***PmAlx1*is expressed in the sea star epithelial mesoderm, but induces skeletogenesis in sea urchins.** WMISH using a probe against *PmAlx1* demonstrates that transcripts are localized to the central vegetal pole mesoderm of sea star blastulae (**A**); in later development, transcripts are detected in the coelomic mesoderm, but never in the mesenchyme, as seen by two-color *in situ* hybridization with *PmEts1* (**B**). In larvae, staining is detected in the lateral aspects of the anterior coeloms (filled arrows in **C**) as well as in the posterior coelom (arrow in C). To confirm that the absence of a larval skeleton in sea stars is not due to a difference in the sea star PmAlx1 protein, we overexpressed *PmAlx1* in sea urchins. (**D-F**) Ectopic expression of *PmAlx1* mRNA in sea urchin embryos results in increased skeletogenesis, as has been observed for overexpression of sea urchin *alx1* mRNA [41]. Control embryos were injected with mRNA encoding the RFP variant mCherry. Representative sea urchin gastrula are shown (D-F). Ectopic skeletal spicules were observed upon overexpression of *PmAlx1* (E, F). Numbers of embryos showing the illustrated phenotype is shown in the lower right corner (from a total of 40 embryos for the control RFP, and 80 embryos in the PmAlx1 injected embryos); D shows normal skeletal formation, E shows increase in number of skeletal forming centers, and F and dramatic increase in skeleton.

In contrast, *in situ* hybridization (Figure 3A-C) reveals that in sea stars, *PmAlx1* is expressed throughout the vegetal pole mesoderm of blastulae. As development proceeds, *PmAlx1* is continually expressed in mesodermal derivatives. In gastrulae, signal is detected in the mesodermal bulb at the tip of the archenteron. In larvae, *PmAlx1* is expressed in the lateral margins of the coelomic pouches, which are derived from the mesodermal bulb of the gastrula, and in the posterior coelom. Throughout development, this pattern is in strict contrast to what is seen in the sea urchins and sea cucumbers. Taken together, these data suggest that changes in both the regulation and function of *alx1* underlie the evolution of the novel echinoderm skeletogenic lineage. To confirm that a change in the Alx1 protein itself did not contribute to the evolution of larval skeletogenesis, we overexpressed the sea star ortholog of Alx1, *PmAlx1*, in sea urchin embryos. *PmAlx1* overexpressing embryos, but not embryos with ectopic control RNA (mCherry), form ectopic skeletal elements (Figure 3D-F). This is consistent with the phenotype associated with overexpression of sea urchin *alx1*[41], which suggests that a protein-level change in Alx1 did not cause the evolution of larval skeletogenesis.

### Exclusion of TF expression from the sea cucumber SM during gastrulation reveals additional similarities with the sea urchin skeletogenic lineage

**Figure 4:**
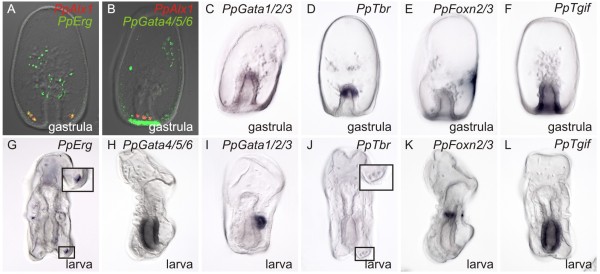
**The skeletogenic centers of sea cucumbers do not express*gata4/5/6*,*gata1/2/3*,*tbr*,*tgif*or*foxn2/3.*** WMISH performed against gastrula stage embryos (**A-F**) show localization of only *PpErg* and *PpAlx1* transcripts in the skeletogenic mesenchyme (**A**). A probe designed against *PpGata4/5/6* is localized to other mesenchyme cells (**B**). No other factor identified in this study showed expression in mesenchyme. WMISH with probes antisense to *PpGata1/2/3* and *PpTbr* are showed localized staining at the tip of the archenteron in gastrulae (**C**, **D**), while *PpFoxn2/3* antisense probes stain only within the ectoderm (**E**) and those antisense to *PpTgif* in the endoderm (**F**). In larvae, *PpErg* antisense probes are localized to the SM (**G**); and see higher magnification in inset. Probes against *PpGata4/5/6* stain within the midgut (**H**), while those against *PcGata1/2/3* (**I**) are present in the posterior coelom. No staining is found for *PpTbr* at this stage (**J**), see higher magnification of SM cells in insert that show no staining. Probes against *PpFoxn2/3* (**K**) ultimately show localization in the posterior foregut. *PpTgif* antisense probes are localized to the midgut of larvae (**L**).

The presence of an SM lineage in both sea urchin and sea cucumber embryos provides an opportunity to begin to uncover the minimal complement of regulatory genes required for skeleton deposition. Therefore, we next carefully examined the expression of regulatory genes within the newly discovered sea cucumber SM after ingression. Two-color *in situ* hybridization clearly shows that in sea cucumber gastrulae, while *PpAlx1* and *PpGata4/5/6* do not localize to the same cells, a subset of *PpErg*^+^ cells co-express *PpAlx1* (Figure 4A, B). Similarly, in larvae, only *PpErg* is expressed in the dorsal cluster of skeletogenic cells (Figure 4G). No other TFs we identified are expressed in the mesenchyme (Figure 4C-F, H-M). A previous study suggests that a sea cucumber *ets1* ortholog is also expressed in the spicule forming cells of a related sea cucumber [39]. The expression of *alx1*, *erg* and *ets1*, but not *gata1/2/3* and *gata4/5/6* expression in these cells is similar to the regulatory state of the sea urchin SM during blastula stages in sea urchins. In contrast, SpTbr is needed for skeleton deposition in sea urchins [19]. The fact that *PpTbr* is not expressed in the sea cucumber SM suggests that the role of SpTbr in larval skeletogensis is an apomorphy of modern sea urchins. Although less parsimonious, it is also possible that *tbr* expression was secondarily lost from the sea cucumber SM.

## Conclusions

### The evolution of novelty

A comparison of the regulatory state of embryonic mesenchyme in three classes of echinoderm embryos, those of sea urchins, sea cucumbers and sea stars, provides an extraordinary opportunity to understand the trajectory of change associated with a novel morphology. The sea urchin ingresses skeletogenic, pigment and blastoceolar cells as mesenchyme. Like the sea urchin, the phylogenetically intermediate sea cucumber also ingresses a skeletogenic lineage that is distinct from blastocoelar cells. Thus, the skeletogenic mesenchyme, defined as a population of mesenchyme expressing *alx1* that secretes the larval skeleton, is a synapomorphy of at least sea cucumbers and sea urchins. In the sea cucumber SM, however, this lineage secretes only a very small skeletal element in contrast to the dramatic skeleton that defines the morphology of the sea urchin pluteus larva.

**Figure 5:**
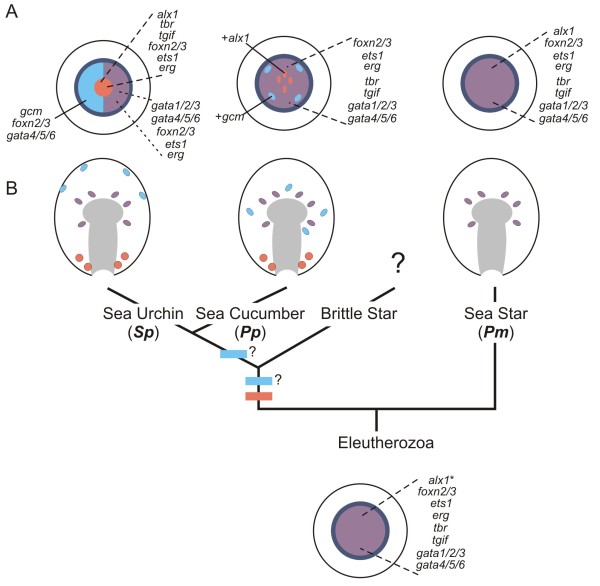
**Model describing regulatory reorganization of the echinoderm mesoderm and evolution of the larval skeletogenic mesenchyme.** (**A**) Regulatory states for the mesoderm of sea urchins, sea cucumbers and sea stars are shown in the top row of the schematic. The dark blue ring represents the endodermal territory that surrounds the mesoderm. Orange regions indicate derived *alx1* expression. Genes expressed in each territory are listed. (**B**) In later development, these territories will form two types of mesenchyme: the *alx1*^+^ SM and the blastocoelar cells (orange and purple, respectively). Gene expression patterns not described in this work have been previously reported [20-30,32-34,39]. Of the organisms studied, only sea cucumbers and sea urchins are known to make skeletogenic mesenchyme, though brittle stars also form a larval skeleton. By comparing gene expression in the mesodermal territories of sea urchins, sea cucumbers and sea stars, we extrapolated the regulatory state of the mesoderm of the echinoderm ancestor (the proto-mesoderm), shown at the base of the tree (phylogeny after [11,14,15]). The broad expression of most TFs within the mesoderm of sea cucumbers and sea stars supports the hypothesis that there was a pan proto-mesoderm during blastula stages. Subsequent regulatory changes within the mesoderm in the lineage leading to at least sea urchins and sea cucumbers created a population of cells, marked by their expression of *alx1*, that go on to form the larval skeleton.

The relatively simple developmental paradigm here allows us reconstruct the ancestral mesodermal regulatory state of the vegetal pole territory in the blastulae of these echinoderms. This was a single mesodermal domain that expressed at least the factors *erg*, *ets1, foxn2/*3*, gata1/2/*3*, gata4/5/*6, *tbr* and *tgif*, and possibly *alx1* (Figure 5). Knowledge of the plesiomorphic state provides the essential framework for understanding how novelty arises. We suggest that the ancestor of the sea cucumber + sea urchin clade may have had a mesoderm with a regulatory state similar to that of extant sea cucumbers and may have developed only a rudimentary larval skeleton. In this scenario, both the segregation of gene expression within the vegetal pole mesoderm and the growth of the larval skeleton have been further elaborated upon in sea urchins. An alternative scenario is that the sea cucumber might also represent a secondary simplification. If the common ancestor of sea cucumbers and sea urchins had separated out expression of, for example, *alx1, tbr* and *tgif* from *gata4/5/6* and *gata1/2/3* as is seen in sea urchins, that segregation would have had to be lost during sea cucumber evolution, returning them to the ancestral state. This would imply that a secondary simplification resulted in extensive reversal to an ancestral state. We find this second scenario unlikely as it violates parsimony. A third scenario is that sea cucumbers ancestrally maintained expression of additional transcription factors in the ingressed SM; for example, like sea urchins, they may have expressed *tgif* and *tbr* in these cells. This greater compliment of regulatory genes might have allowed for more complex morphology of the larva skeleton than extant sea cucumber exhibit. In sea cucumbers, expression of some of these transcription factors may have subsequently been lost from the SM, leading to a secondary reduction in larval skeleton complexity. In any case, the evolutionary novelty of sea urchins was then to further segregate these lineages into a micromere and vegetal plate mesoderm. These data also strongly suggest that the initiation of larval skeletogenesis and the elaboration of the skeleton are separable, both developmentally and evolutionarily. Our data also suggest that while Alx1 is a key transcription factor needed for the formation of the skeletal spicule in sea urchins and sea cucumbers, a change in the protein itself is unlikely to be the evolutionary change needed for the new larval cell type. This implies that rewiring of the GRN downstream of *alx1* led to the expression of genes needed for the larval skeleton.

An understanding of the evolutionary trajectory of echinoderm larval skeleton formation could be strengthened by a molecular study of the embryology of this process in brittle stars. If the third scenario is upheld, parsimony also supports a single origin of the echinoderm larval skeleton and, therefore, a monophyletic grouping of sea urchins, sea cucumbers and brittle stars, with the sea star as the outgroup that forms no skeleton. Molecular phylogenetic analysis finds equal support for this grouping as for there being a separate brittle star + sea star clade [11,14,15].

The other important, and unexpected, observation to arise from this comparison is that there is a loss of gene expression in each of the sea urchin mesodermal lineages relative to the ancestral mesoderm (Figure 5). The sea urchin uses distinct GRNs, linked in part by intercellular signaling, to drive the development of its skeletogenic, pigment and blastocoelar cells. Each of these sub-populations retains some, but not all, features of the ancestral network. Thus, partitioning of an ancestral GRN may have been an important mechanism for allowing evolutionary change.

## Methods

### Culturing and microinjection of embryos

Adult animals were collected off the southern California coast, USA. Spawning was induced in adult sea cucumbers by intra-coelomic injection of 100nM NGLWY-amide followed by heat shock in room temperature sea water after the method of Kato *et al.*[42]. Freshly shed eggs were mixed with dilute sperm to fertilize, and embryos were cultured in artificial seawater at 15°C until the desired stage. Sea star and sea urchin embryos were prepared according to standard protocols [31,43] and cultured at 15°C until the desired stage. Zygotes were injected as previously described for sea urchin and sea star [43,44]. Sea cucumber microinjection was performed following protocols for sea stars.

### Cloning sea cucumber and sea star transcription factor orthologs

RNA was isolated from embryos at blastula, gastrula and larva stages using the Total Mammalian RNA Miniprep kit (Sigma, St. Louis, MO, USA). First strand cDNA synthesis was performed with the iScript Select cDNA Synthesis kit (BioRad, Hercules, CA, USA). Degenerate primers were designed against conserved domains and PCR was performed under non-stringent conditions. Once partial sequences were obtained, 5^′^ and 3^′^ RACE was performed according to directions (GeneRacer; Invitrogen, Carlsbad, CA, USA). PCR primer sequences are available on request. Sequences were deposited in GenBank under the following accession numbers: *PmAlx1*, GenBank:JQ740823; *PpAlx1*, GenBank:JQ740824; *PpErg*, GenBank:JQ740825; *PpFoxa*, GenBank:JQ740826; *PpFoxn2/3*, GenBank:JQ740827; *PpGata1/2/3*, GenBank:JQ740828; *PpGata4/5/6*, GenBank:JQ740829; *PpGcm*, GenBank:JQ740830; *PpTbr*, GenBank:JQ740831; *PpTgif*, GenBank:JQ740832.

### Whole mount in situ hybridization (WMISH)

Antisense digoxigenin-labeled RNA probes were generated against genes of interest. WMISH was performed as previously described for sea stars [34]. Embryos were photographed using DIC optics on a Leica DMI4000B at 200× magnification using the Leica Application Suite software (Leica, Wetzlar, Germany).

### Two-color in situ hybridization

Antisense dinitrophenol-labeled RNA probes were generated against *PpAlx1*, *PpFoxa*, *PpGcm*, and *PmEts1*. Embryos were fixed as for WMISH, and two-color WMISH was carried out as described by Yankura *et al.*[45]. In some cases, embryos were counterstained with 1 μM DAPI. Embryos were photographed on a LSM 510 Meta/UV DuoScan Inverted Spectral Confocal Microscope with the ZEN 2009 Imaging suite (Carl Zeiss, Thornwood, NY, USA).

### RNA overexpression of *PmAlx1* and RFP mRNA in sea urchins and knockdown of PpAlx1

Full-length *PmAlx1* and *mCherry*, an RFP derivative, were cloned into the pCS2+ vector. The resulting plasmids were linearized with HpaI and NotI, respectively. mRNA was synthesized using the mMessage mMachine kit (Ambion; Grand Island, NY, USA). The PpAlx1 morpholino antisense oligonucleotide (MASO) 5^′^ GGCTCACAAAGTCTGAAATAATCAT 3^′^ was designed by GeneTool, LLC (Philomath, O, USA). The mRNAs and MASO were injected into embryos in 200 mM KCl containing 0.5 mg/mL fluorescent rhodamine tracer.

## Abbreviations

GRN: Gene regulatory network; SM: Skeletogenic mesenchyme; WMISH: Whole mount *in situ* hybridization.

## Competing interests

The authors declare that they have no competing interests.

## Authors’ contributions

VFH and BSM designed the research. CE worked on overexpressing *PmAlx1* in sea urchins. CK cloned *PmAlx1*. EPW helped develop methods for working with sea cucumbers and assessed gene expression in sea cucumber embryos. BSM aided with all the above and performed microinjections in sea urchins and sea cucumbers. VFH and BSM analyzed data and wrote the paper. All authors read and approved the final manuscript

## Supplementary Material

Additional file 1 **Development of the sea cucumber,*Parastichopus parvimensis.*** (**A**) As in sea stars, early cleavage of *P. parvimensis* is equal and little cell-cell adhesion is seen between blastomeres (see space between arrows). Divisions are not synchronous. Prior to gastrulation, the embryos elongate along the animal-vegetal axis, and a thickening is observed at the vegetal pole. (**B**) Mesenchyme ingresses from the vegetal pole (arrow) before invagination of the archenteron occurs. (**C**) While most mesenchyme remains associated with the tip of the archenteron during early gastrulation, a few cells migrate to take up a position near the blastopore (arrows). (**D**) At the mid-gastrula stage around 48 hpf, the mesenchyme has begun to migrate, and additional mesenchymal cells ingress from the archenteron. (**E, F**) By 3 days of development, the mouth has formed, and the embryo can now be considered an auricularia larva. he archenteron has differentiated into morphologically distinct fore-, mid-, and hindgut regions, with presumptive circumesophageal muscle cells seen along the edges of the foregut. A posterior coelom is evident near to the left of the midgut (open arrow in **F**), but there are no obvious anterior coeloms. A small skeletal spicule is evident near the anus (closed arrow in **F**). (**G, H**) The auricularia further elaborates over time, and by 6 days of development the archenteron has differentiated into morphologically distinct fore (F)-, mid (M)-, and hindgut (H) regions. A small posterior skeletal spicule can be clearly observed (arrow in **H**). Hpf - hours post fertilization; d - days post fertilization. Click here for file

Additional file 2 **Phylogenetic analysis confirms orthology of the newly identified sea cucumber and sea star transcription factors.** Phylogenies of the ETS (**A**), GATA (**B**), TBX (**C**), TALE homeodomain (**D**), FOX (**E**), and paired-class homeodomain (**F**) families are shown. Protein sequences were obtained using the BLASTx algorithm against the NCBI non-redundant protein database [46]. Proteins from other families within a larger gene group were also included in the analysis as outgroups. For all partial phylogenies except the paired-class homeodomains, trees were constructed in PAUP using the neighbor-joining method, and confirm the placement of PpErg, PpFoxA, PpFoxN2/3, PpGata1/2/3, PpGata4/5/6, PpTbr and PpTgif as members of their respective families. Bootstrap values are shown for select clades. As comparison of the homeodomain only could not distinguish between echinoderm Alx1 vs. Alx4, additional sequence was analyzed using the BioNJ tool available at Methodes et Algorithmes pour la Bio-informatique [47-49]. This tree, based on a whole protein alignment clearly places the Alx sequences from both the sea cucumber and sea star as members of the Alx1 family. Also shown in panel G are partial alignments of a stretch of amino acids just C-terminal to the homeodomains and the C-terminal OAR domains of echinoderm Alx1 and Alx4 orthologs. The hashmarks above the alignment indicate residues that are identical among the proteins designated Alx1, but are not conserved in SpAlx4; the asterisks below the alignment signify identity across all proteins. This analysis suggests that the Alx1 orthologs isolated from sea cucumbers and sea stars are the true orthologs of SpAlx1, rather than belonging to the Alx4 orthology group. Ap, *Asterina pectinifera*; Bf, *Brachiostoma floridae*; Bl, *Brachoistoma lanceolatum*; Dm, *Drosophila melanogaster*; Dr, *Danio rerio*; Gg, *Gallus gallus*; Hs, *Homo sapiens*; Lv, *Lytechinus variegatus*; Nv, *Netmatostella vectensis*; Pc, *Parastichopus californicus*; Pm, *Patiria miniata*; Sk, *Saccoglossus kowalevskii*; Sp, *Strongylocentrotus purpuratus*; Xl, *Xenopus laevis*; Xt, *Xenopus tropicalis*. Click here for file
