# Supplementary figures and images for: Development of an embryonic skeletogenic mesenchyme lineage in a sea cucumber reveals the trajectory of change for the evolution of novel structures in echinoderms
Source: EvoDevo. 2012 Aug 9;3:17. doi: 10.1186/2041-9139-3-17 (PMC3482387; doi:10.1186/2041-9139-3-17)

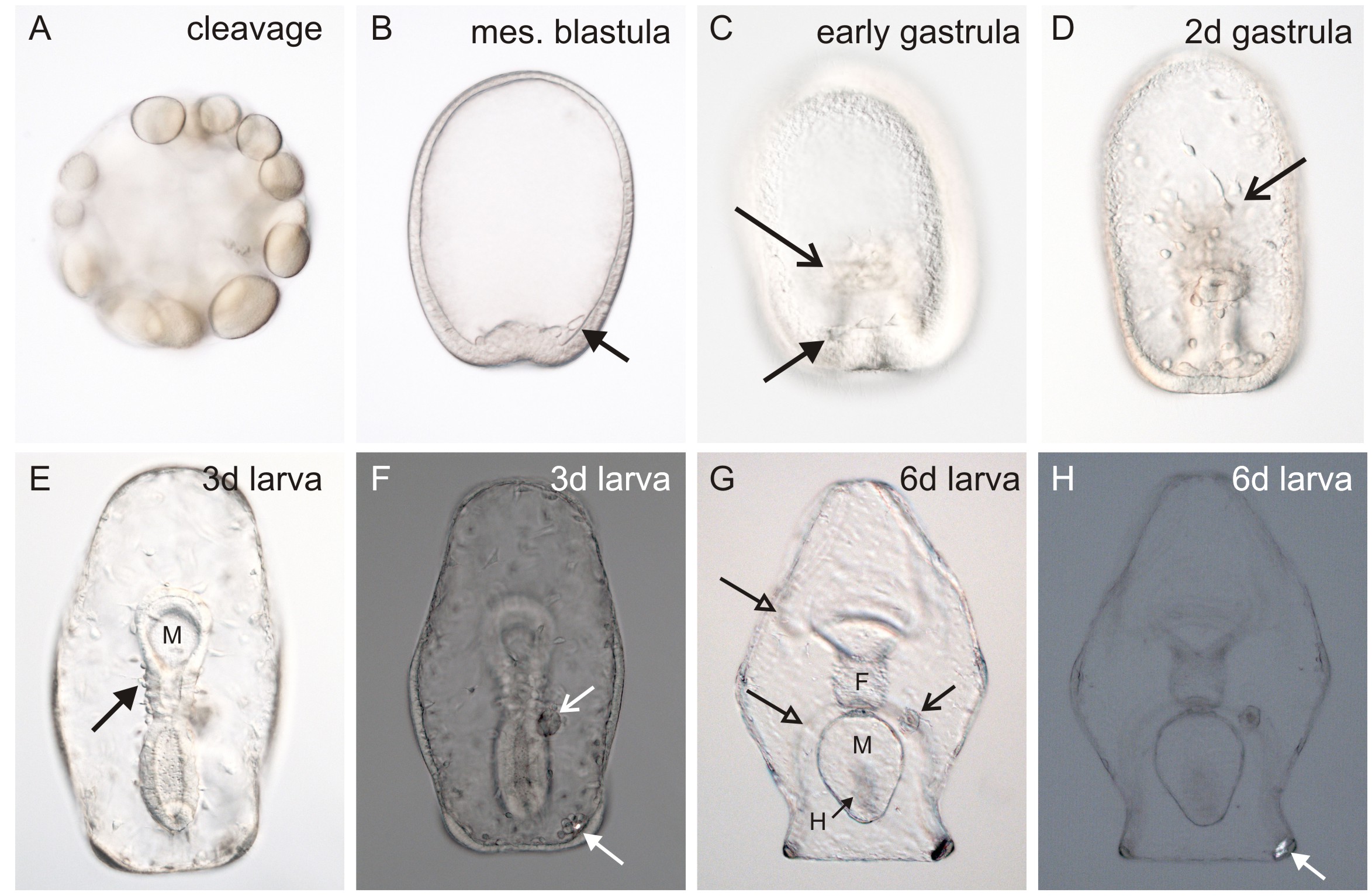

Supplement: Additional file 1 — Development of the sea cucumber,Parastichopus parvimensis. (A) As in sea stars, early cleavage of P. parvimensis is equal and little cell-cell adhesion is seen between blastomeres (see space between arrows). Divisions are not synchronous. Prior to gastrulation, the embryos elongate along the animal-vegetal axis, and a thickening is observed at the vegetal pole. (B) Mesenchyme ingresses from the vegetal pole (arrow) before invagination of the archenteron occurs. (C) While most mesenchyme remains associated with the tip of the archenteron during early gastrulation, a few cells migrate to take up a position near the blastopore (arrows). (D) At the mid-gastrula stage around 48 hpf, the mesenchyme has begun to migrate, and additional mesenchymal cells ingress from the archenteron. (E, F) By 3 days of development, the mouth has formed, and the embryo can now be considered an auricularia larva. he archenteron has differentiated into morphologically distinct fore-, mid-, and hindgut regions, with presumptive circumesophageal muscle cells seen along the edges of the foregut. A posterior coelom is evident near to the left of the midgut (open arrow in F), but there are no obvious anterior coeloms. A small skeletal spicule is evident near the anus (closed arrow in F). (G, H) The auricularia further elaborates over time, and by 6 days of development the archenteron has differentiated into morphologically distinct fore (F)-, mid (M)-, and hindgut (H) regions. A small posterior skeletal spicule can be clearly observed (arrow in H). Hpf - hours post fertilization; d - days post fertilization. [file 2041-9139-3-17-S1.doc]

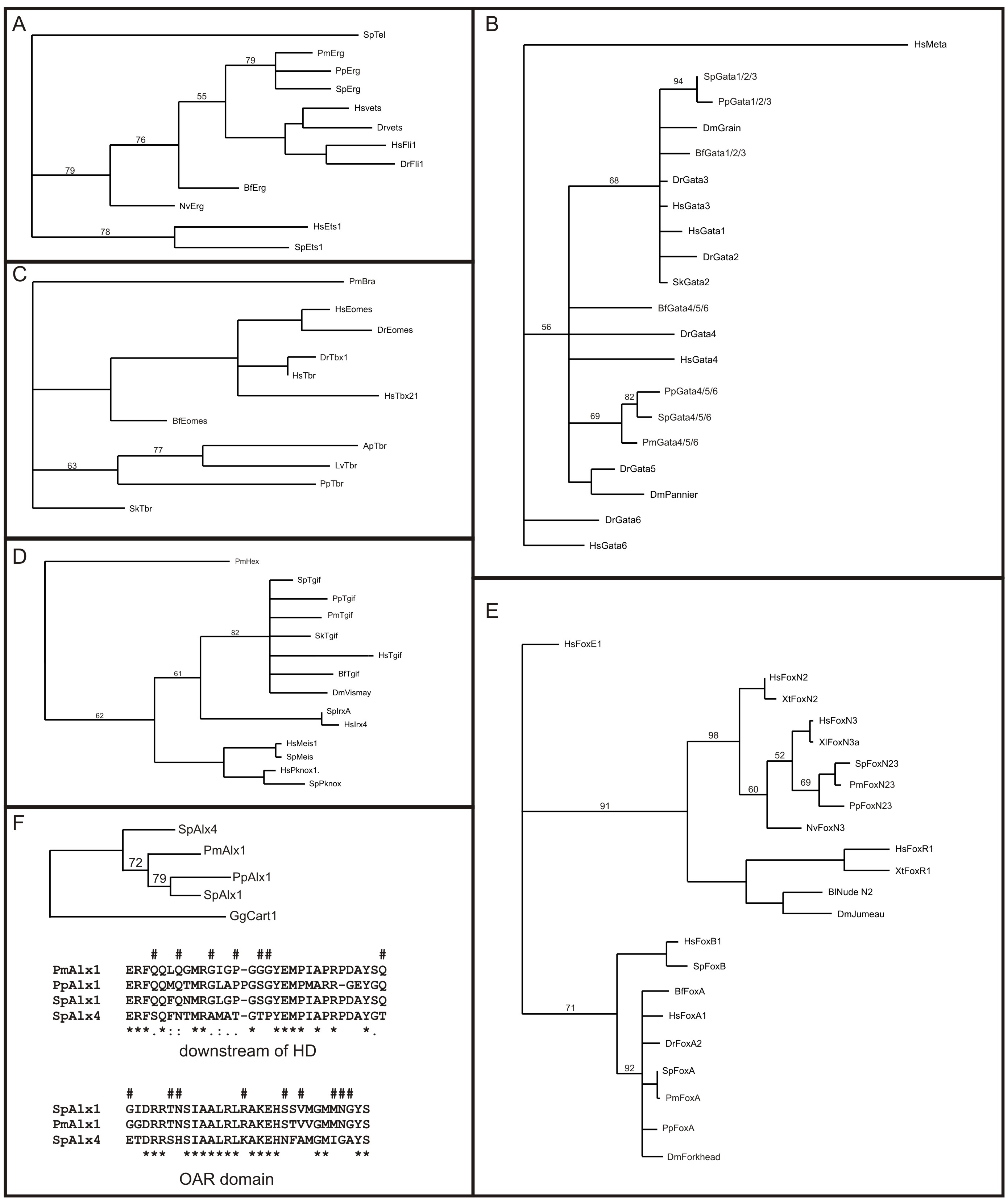

Supplement: Additional file 2 — Phylogenetic analysis confirms orthology of the newly identified sea cucumber and sea star transcription factors. Phylogenies of the ETS (A), GATA (B), TBX (C), TALE homeodomain (D), FOX (E), and paired-class homeodomain (F) families are shown. Protein sequences were obtained using the BLASTx algorithm against the NCBI non-redundant protein database [46]. Proteins from other families within a larger gene group were also included in the analysis as outgroups. For all partial phylogenies except the paired-class homeodomains, trees were constructed in PAUP using the neighbor-joining method, and confirm the placement of PpErg, PpFoxA, PpFoxN2/3, PpGata1/2/3, PpGata4/5/6, PpTbr and PpTgif as members of their respective families. Bootstrap values are shown for select clades. As comparison of the homeodomain only could not distinguish between echinoderm Alx1 vs. Alx4, additional sequence was analyzed using the BioNJ tool available at Methodes et Algorithmes pour la Bio-informatique [47-49]. This tree, based on a whole protein alignment clearly places the Alx sequences from both the sea cucumber and sea star as members of the Alx1 family. Also shown in panel G are partial alignments of a stretch of amino acids just C-terminal to the homeodomains and the C-terminal OAR domains of echinoderm Alx1 and Alx4 orthologs. The hashmarks above the alignment indicate residues that are identical among the proteins designated Alx1, but are not conserved in SpAlx4; the asterisks below the alignment signify identity across all proteins. This analysis suggests that the Alx1 orthologs isolated from sea cucumbers and sea stars are the true orthologs of SpAlx1, rather than belonging to the Alx4 orthology group. Ap, Asterina pectinifera; Bf, Brachiostoma floridae; Bl, Brachoistoma lanceolatum; Dm, Drosophila melanogaster; Dr, Danio rerio; Gg, Gallus gallus; Hs, Homo sapiens; Lv, Lytechinus variegatus; Nv, Netmatostella vectensis; Pc, Parastichopus californicus; Pm, Patiria miniata; Sk, Saccogl [file 2041-9139-3-17-S2.doc]
